# Integrative Multi-Omics Analysis Identifies Transmembrane p24 Trafficking Protein 1 (TMED1) as a Potential Prognostic Marker in Colorectal Cancer

**DOI:** 10.3390/biology13020083

**Published:** 2024-01-29

**Authors:** Xin Guo, Wei Zhou, Jinmei Jin, Jiayi Lin, Weidong Zhang, Lijun Zhang, Xin Luan

**Affiliations:** 1 School of Pharmacy, Shanghai Jiao Tong University, Shanghai 200240, Chinawdzhangy@hotmail.com (W.Z.); 2 Institute of Interdisciplinary Integrative Medicine Research, Shanghai University of Traditional Chinese Medicine, Shanghai 201203, China; 3 Shanghai Institute of Pharmaceutical Industry, China State Institute of Pharmaceutical Industry, Shanghai 201203, China; 4 School of Pharmacy, Second Military Medical University, Shanghai 200433, China

**Keywords:** multi-omics, TMED1, colorectal cancer, prognosis

## Abstract

**Simple Summary:**

In this study, the expression and prognostic significance of the transmembrane p24 trafficking protein 1 (TMED1) in colorectal cancer were investigated by utilizing patient survival data and multi-omics datasets, including immunohistochemical staining, transcriptomics, and proteomics. The results indicated that TMED1, functioning as an oncogene, is upregulated in colorectal cancer and exhibits a significant association with an unfavorable prognosis. Furthermore, the downregulation of TMED1 was found to impact the cell cycle and apoptotic signaling pathways. Additionally, a positive correlation was identified between TMED1 and other members of the TMED family (TMED2, TMED4, TMED9, and TMED10) in colorectal cancer. The protein–protein interaction network analysis further indicated the potential influence of TMED1 on immune regulation. Consequently, TMED1 emerged as a promising candidate biomarker for assessing the progression and prognosis of colorectal cancer.

**Abstract:**

Several TMED protein family members are overexpressed in malignant tumors and associated with tumor progression. TMED1 belongs to the TMED protein family and is involved in protein vesicular trafficking. However, the expression level and biological role of TMED1 in colorectal cancer (CRC) have yet to be fully elucidated. In this study, the integration of patient survival and multi-omics data (immunohistochemical staining, transcriptomics, and proteomics) revealed that the highly expressed TMED1 was related to the poor prognosis in CRC. Crystal violet staining indicated the cell growth was reduced after knocking down TMED1. Moreover, the flow cytometry results showed that TMED1 knockdown could increase cell apoptosis. The expression of TMED1 was positively correlated with other TMED family members (TMED2, TMED4, TMED9, and TMED10) in CRC, and the protein–protein interaction network suggested its potential impact on immune regulation. Furthermore, TMED1 expression was positively associated with the infiltration levels of regulatory T cells (Tregs), cancer-associated fibroblasts (CAFs), and endothelial cells and negatively correlated with the infiltration levels of CD4+ T cells, CD8+ T cells, and B cells. At last, the CTRP and GDSC datasets on the GSCA platform were used to analyze the relationship between TMED1 expression and drug sensitivity (IC_50_). The result found that the elevation of TMED1 was positively correlated with IC_50_ and implied it could increase the drug resistance of cancer cells. This research revealed that TMED1 is a novel prognostic biomarker in CRC and provided a valuable strategy for analyzing potential therapeutic targets of malignant tumors.

## 1. Introduction

Colorectal cancer (CRC) has emerged as the third most commonly diagnosed and second most deadly cancer worldwide [1,2]. Obesity, low physical exercise, and smoking could increase the risk of CRC [3,4,5]. Despite the advancements in surgery, radiotherapy, and chemotherapy, the five-year survival rate of advanced and metastatic CRC is still below 20% [6,7]. In addition, the targeted therapy of CRC patients is still unsatisfactory due to the high tumor heterogeneity, late-stage diagnosis, and drug resistance [8,9,10]. Therefore, it is urgent to discover novel molecular targets that can facilitate personalized diagnosis and treatment, ultimately enhancing patient prognosis.

Transmembrane p24 trafficking protein 1 (TMED1) belongs to the TMED protein family, also known as the p24 family, and plays a part in protein vesicular trafficking [11,12]. As the critical regulator of protein trafficking along the secretory pathway, the TMED proteins influence the composition, structure, and function of the endoplasmic reticulum and the Golgi apparatus [12,13]. Disruption of the secretory pathway could affect cell differentiation and survival, ultimately resulting in disease [14]. There are nine proteins that make up the four subfamilies of the TMED family: α (TMED 4, 9), β (TMED 2), γ (TMED 1, 3, 5, 6, 7), and δ (TMED 10) [15]. The development of various types of malignancies is closely linked to specific subfamilies, including the α subfamily (associated with breast cancer, epithelial ovarian carcinoma, colon cancer, hepatocellular carcinoma), the β subfamily (related to head and neck squamous cell carcinoma, lung cancer, breast cancer, ovarian cancer, squamous cervical carcinoma), the γ subfamily (associated with glioblastoma, lung cancer, breast cancer, epithelial ovarian carcinoma), and the δ subfamily (related to head and neck squamous cell carcinoma, prostate cancer) [15]. Furthermore, most members of this family have been reported to be associated with poor prognosis [16,17]. The expression of TMED2 is elevated in head and neck squamous carcinoma and breast cancer and acts as an unfavorable prognostic biomarker [18,19]. TMED3 is highly expressed in malignant melanoma and lung squamous cell carcinoma, which is important in advancing cancer [20,21]. TMED5 is related to the clinical stage, and it could promote cell proliferation, migration, and invasion in cervical cancer and interact with WNT7B to activate the WNT-β-catenin signaling pathway [22]. TMED9 promotes colon cancer metastases and relates to vascular invasion and poor prognosis in hepatocellular carcinoma [23,24]. TMED1 is highly expressed in multiple tumor tissues [18], but its prognostic significance in malignant tumors has yet to be clarified, especially in CRC.

Multi-omics techniques such as genomics, transcriptomics, and proteomics were widely used to identify new therapeutic targets and prognostic biomarkers of tumors [25,26]. Multi-omics data contributed to a more comprehensive understanding and annotation of gene functions [27]. HKDC1 and SLC16A3 were identified as potential prognostic biomarkers of intrahepatic cholangiocarcinoma using multi-omics methods [28]. Whole exome sequencing, RNA sequencing, proteome profiling, and phosphoproteome profiling were used to identify TCN1 as a potential cholangiocarcinoma prognostic biomarker [29]. HSP 90β was identified as a potential prognostic biomarker of lung adenocarcinoma using data from the genome, transcriptome, proteome, and phosphoproteome [30]. The present study first clarified TMED1 expression and its prognostic value in CRC based on multi-omics data and experimental validation. The elevated TMED1 expression indicated an unfavorable prognosis and an aggressive phenotype in CRC. Furthermore, the association between TMED1 expression and immune infiltration was analyzed in the CRC. These findings propose that TMED1 could serve as a novel prognostic biomarker and therapeutic target for CRC.

## 2. Materials and Methods

### 2.1. Patient Information and the Expression of TMED1

To analyze the gene expression levels of TMED1 in tumor and normal tissues, the Cancer Genome Atlas Program (TCGA) and Genotype-Tissue Expression (GTEx) [31] databases on the Gene Expression Profiling Interactive Analysis (GEPIA) platform [32] were employed. GEPIA is an online platform designed for analyzing the expression data from 9736 tumors and 8587 normal samples obtained from the TCGA and GTEx projects [32]. The cohort (GSE110223 microarray dataset) was obtained from the Gene Expression Omnibus (GEO) database to perform gene expression profiling in the CRC patients’ paired tumor and normal tissue. The Clinical Proteomic Tumor Analysis Consortium (CPTAC) datasets on the University of Alabama at Birmingham cancer data analysis portal (UALCAN) [33] platform were utilized to perform the protein expression levels of TMED1. UALCAN is a user-friendly and interactive web resource that provides a comprehensive analysis of cancer omics data. For each query, the database offers graphs and plots that illustrate the protein expression profile in both normal and tumor tissues. It will also provide statistical significance by comparing data between the normal group and the tumor group. Furthermore, the Human Protein Atlas (HPA) [34] was used to assess the protein expression of TMED1 in both normal and tumor tissues. When TMED1 is searched on the HPA platform, the expression of the protein in cells, tissues, and organs will be obtained. A total of 434 gene profiles (383 tumor samples and 51 normal samples) of CRC were acquired from the UCSC Xena [35]. Moreover, corresponding clinical information, including primary tumor sites, T histopathologic stage, and survival condition, was obtained for analysis. R packages “ggplot2 (version 3.4.3)” and “ggalluvial (version 0.12.4)” [36] were used to draw a Sankey diagram to describe the relationships among the TMED1 expression level, clinical pathological information, and survival status.

### 2.2. Survival Analysis

The Kaplan–Meier Plotter tool [37] was utilized to characterize the prognostic significance of TMED1 expression in CRC patients. When the “Auto select best cutoff” checkbox was selected from the Kaplan–Meier Plotter tool, the system calculated all potential cutoff values within the lower and upper quartiles. After that, the threshold that performed the best was the final cutoff value [37]. The patients were categorized into high and low-expression groups based on the best cutoff of TMED1 mRNA expression. The log-rank test was employed to compare the overall survival (OS) and relapse-free survival (RFS) rates between the groups with low and high expression levels of TMED1. The significance level was set at a *p*-value of less than 0.05.

### 2.3. Protein–Protein Interaction (PPI) Network and Reactome Pathway Analysis

To construct a protein–protein interaction (PPI) network of co-expression genes, the Search Tool for the Retrieval of Interacting Genes/Proteins (STRING, version 11.5) database [38] with an interaction score higher than 0.4 was utilized. The STRING database is a comprehensive resource that provides information on both known and predicted protein–protein interactions. The functionality of the STRING database allows for a comprehensive exploration of protein–protein interactions and their roles in various cellular processes. The Spearman’s correlation analysis between TMED1 and co-expression genes was performed using the GEPIA portal. The Reactome pathway database [39] was employed to explore the enrichment pathway analysis of co-expression genes. The “ggplot2” R package was applied for the top 20 Reactome pathway visualization, according to the false discovery rate (FDR).

### 2.4. Immune Infiltration Analysis

The TIMER 2.0 tool [40] utilized the “immunedeconv” R package with six algorithms, including TIMER [41], CIBERSORT [42], quanTIseq [43], xCell [44], MCP-counter [45], and EPIC [46]. These algorithms were discovered to possess distinct properties and strengths. Different tissue types may induce different intrinsic expressions of cancer cells and produce different immune environments [47]. TIMER is the only method that takes tissue specificity into account when estimating the number of immune cells. TIMER can estimate six immune cell types, while xCell can estimate more immune cell types. MCP-counter is an algorithm used to estimate immune cell infiltration in tumor tissue by analyzing gene expression. It can evaluate the presence of multiple immune-related cell types. CIBERSORT is capable of deconvolving more intricate subsets of T cell signatures, providing a more detailed analysis. EPIC and quanTIseq have the advantage of being able to generate scores directly, which are interpreted as cell fractions. In order to obtain reliable immune infiltration estimations, the above algorithms were used for the analysis. The presence of tumor infiltrating is a crucial factor in determining the effectiveness of cancer treatment and predicting patient prognosis [48]. TIMER 2.0 was applied to perform the TMED1 expression and the immune infiltration. It serves as a comprehensive resource for investigating the correlation between infiltrating immune cells and genes across a diverse range of cancer types. *p* < 0.05 was used as the criterion for determining the correlation between TMED1 expression and the infiltration levels of immune cells. In addition, the “ggplot2 (version 3.4.3)” R package was used to enhance and visualize the significant immune cells under different algorithms.

### 2.5. Gene Set Variation Analysis (GSVA) and Drug Sensitivity Analysis

The Gene Set Cancer Analysis (GSCA) platform [49] was applied to determine the pathway activity of the co-expression genes. Additionally, the drug sensitivity (IC_50_) was assessed using the Cancer Therapeutics Response Portal (CTRP) and Genomics of Drug Sensitivity in Cancer (GDSC) datasets [50,51]. The IC_50_ of 481 small molecules in 1001 cell lines and 265 small molecules in 860 cell lines from CTRP and GDSC, respectively, and their corresponding mRNA gene expression were collected. Pearson correlation analysis was utilized to examine the relationship between TMED1 mRNA expression and drug IC_50_. The *p*-value was adjusted by FDR.

### 2.6. Cell Culture and RNA Interference

Human colorectal cancer cell lines (HCT 116 and RKO) were acquired from the Cell Bank of the Shanghai Institute of Cell Biology. The HCT 116 cells were cultured in McCoy’s 5A medium (Gibco, Carlsbad, CA, USA) with 10% fetal bovine serum (FBS) and 1% penicillin–streptomycin mixture added to the above medium. The RKO cells were maintained in MEM with NEAA (Meilun Biotechnology, Dalian, China) supplemented with 10% FBS and 1% penicillin–streptomycin mixture. The cells were cultured in a 37 °C incubator (Thermo Fisher, Waltham, MA, USA) with 5% CO_2_. The synthesis of small interfering RNA (siRNA) was entrusted to GenePharma (Shanghai, China). The cells were placed into 6-well plates (1.5 × 10^5^ cells/well), and the siNC group was transfected with the scrambled siRNA (negative control), and the siTMED1 group was transfected with the siRNA specific for TMED1 (Table S1). All siRNA transfections were performed with GP-transfect-Mate at a 75 nM final concentration following the manufacturer’s protocol. The transfected cells were collected for the following assays 48 h after transfection. Gene knockdown efficiency was determined by real-time quantitative PCR.

### 2.7. Real-Time Quantitative PCR

RNA extraction from cells was performed using a total RNA extractor (Trizol, B511311, Sangon Biotech, Shanghai, China) according to the manufacturer’s protocol. The HiScript II Q RT SuperMix for qPCR Kit (R223, Vazyme, Nanjing, China) was utilized to reverse transcribe the extracted total RNA into first-strand cDNA. Subsequently, the cDNAs were used for real-time quantitative PCR on a LightCycler 96 Touch Real-Time Quantitative PCR System (Roche, Pleasanton, CA, USA) using ChamQ Universal SYBR qPCR Master Mix (Q711, Vazyme). GAPDH served as the internal control. The primer sequence used for qPCR analyses is shown in Table S2. The 2^−ΔΔCt^ method was employed for determining the relative quantification of gene expression.

### 2.8. Cell Proliferation Assay

#### 2.8.1. Crystal Violet Assay

HCT 116 cells (1.5 × 10^5^ cells/well) were placed into 6-well plates (Corning, Corning, NY, USA) overnight and transfected with the siRNA for 48 h. After removing the supernatant, the cells were washed with phosphate-buffered saline (PBS). Then, the cells were fixed for 30 min using a 4% paraformaldehyde solution, followed by 15 min staining with crystal violet. The Cytation 5 microplate reader system (BioTek, Winooski, VT, USA) was used to capture images and then measure the absorbance at 595 nm after dissolving the cells in a 10% acetic acid solution.

#### 2.8.2. CCK-8 Assay

Transfected CRC cells were seeded into 96-well plates (Corning) at a density of 2.5 × 10^4^ cells/mL in a volume of 200 μL. After incubating overnight, the cell viability was detected by Cell-Counting Kit-8 (CCK-8) every day for four/five days. A total of 10% CCK8 solution was added to each well, and the cells were incubated for 2 h. Then, the OD values (450 nm) were measured using a Cytation 5 microplate reader (BioTek).

### 2.9. Annexin V Apoptosis Assay

The Annexin V-FITC apoptosis assay kit (abs50001, absin, Shanghai, China) was employed to detect cell apoptosis. Briefly, the siRNA-transfected cells were collected and washed with pre-cooled PBS. The cells were transferred into the binding buffer and incubated with 5 μL Annexin V-FITC for 15 min at room temperature in darkness. Then, 5 μL propidium iodide (PI) was added, and the cells were subjected to analysis using flow cytometry (Beckman Coulter, Brea, CA, USA).

### 2.10. Cell Cycle Analysis

The cell cycle detection kit (KGA512, KeyGEN BioTECH, Nanjing, China) was employed to perform cell cycle analysis. After transfecting the cells with siRNA, they were collected and fixed with 70% ethanol overnight at 4 °C. After being washed twice with PBS, the cells were incubated with a mixture of PI and RNase A in a 9:1 (*v/v*) ratio for 30 min at room temperature in darkness. Subsequently, flow cytometry (Beckman Coulter) was employed for the analysis of the cells.

### 2.11. Statistical Analysis

R (version 4.3.1) software was used for data analysis and visualization. The Wilcoxon test was employed to analyze continuous data. Overall survival was analyzed using Kaplan–Meier plots along with the log-rank test. As for the correlation analysis between TMED1 and co-expression genes and the expression of TMED1 and immune infiltration, Spearman’s correlation coefficients were used. Pearson’s correlation analysis was performed to determine the correlation between TMED1 expression and the drug IC_50_. Results were presented as mean ± standard deviation (SD) (bar plot). In all statistical analyses conducted, a significance level of *p* < 0.05 was used to define statistical significance.

## 3. Results

### 3.1. TMED1 Was Highly Expressed in CRC

**Figure 1:**
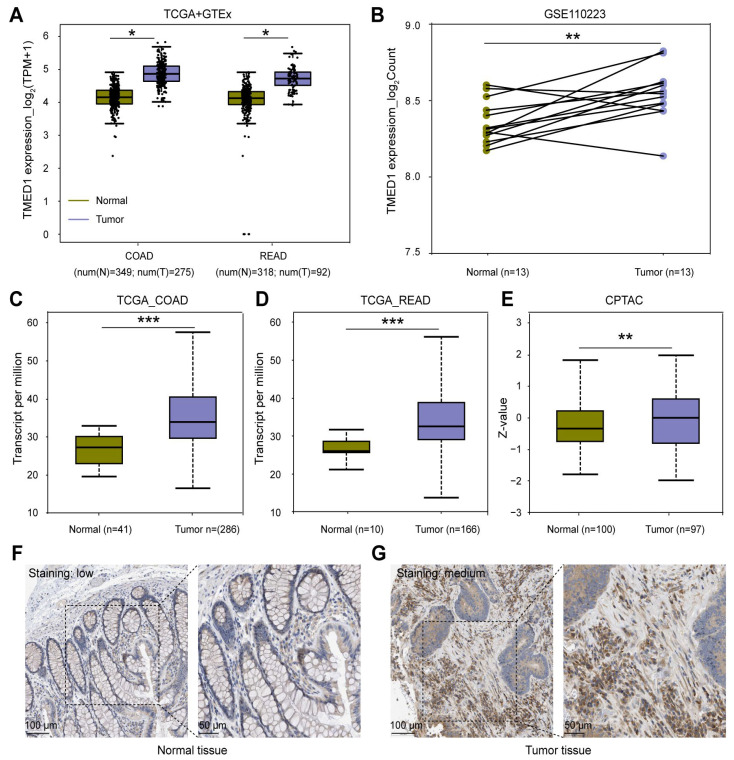
TMED1 was highly expressed in CRC. (**A**) The mRNA expression of TMED1 in tumor and normal tissues of COAD and READ patients from the TCGA and GTEx databases. (**B**) The mRNA expression of TMED1 in paired CRC patients from the GEO database. (**C**,**D**) The mRNA expression of TMED1 in COAD and READ from the TCGA database. (**E**) The protein expression of TMED1 in CRC patients from the CPTAC database. (**F**,**G**) The protein expression of TMED1 in normal tissue and CRC tumor from the HPA database. Boxplots show the median (central line), upper and lower quartiles (box limits), and min-to-max range. (*, *p* < 0.05; **, *p* < 0.01; ***, *p* < 0.001).

To assess the mRNA and protein expression of TMED1 in CRC, data from the TCGA, GEO, GTEx, and CPTAC datasets were collected. According to the results depicted in Figure 1A, the mRNA expression of TMED1 was found to be significantly higher in colon adenocarcinoma (COAD) and rectum adenocarcinoma (READ) compared to normal tissues (*p* < 0.05). The above results were supported by the expression of TMED1 in primary adenocarcinomas and matched normal samples (GSE110223) (*p* < 0.01, Figure 1B). Based on the TCGA data, there was a significant increase in the transcriptional level of TMED1 in COAD and READ when compared to normal tissues (*p* < 0.001, Figure 1C,D). Additionally, the protein expression of TMED1 was analyzed in both normal colon tissues and COAD tissues using the CPTAC datasets and Human Protein Atlas (HPA) immunohistochemistry staining data. It indicated that the protein level of TMED1 was elevated in tumor samples as opposed to samples from normal tissues (*p* < 0.01, Figure 1E–G).

### 3.2. TMED1 Predicted a Poor Prognosis in CRC

**Figure 2:**
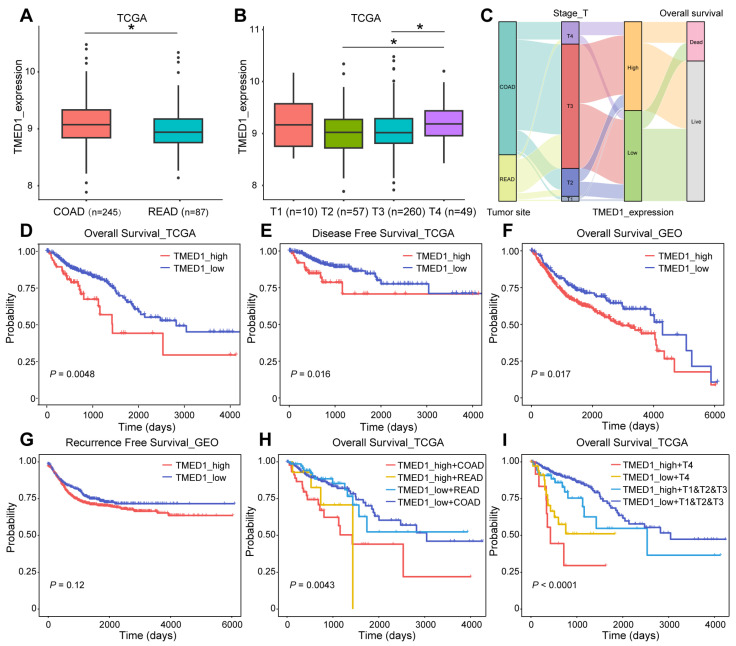
TMED1 predicted a poor prognosis for CRC. (**A**) Analyses for the expression of TMED1 in COAD and READ. (**B**) Analyses for the expression of TMED1 in different T stages. (**C**) A Sankey diagram illustrating the connection between primary tumor sites, T histopathologic stage, the expression of TMED1, and survival status. (**D**) The Kaplan–Meier plot of overall survival in TCGA. (**E**) The Kaplan–Meier plot of disease-free survival in TCGA. (**F**) The Kaplan–Meier plot of overall survival in GEO. (**G**) The Kaplan–Meier plot of recurrence-free survival in GEO. (**H**) The Kaplan–Meier analysis of overall survival of COAD or READ in TCGA. (**I**) The Kaplan–Meier plot of overall survival of T4 or T1 and T2 and T3 in TCGA. Boxplots show the median (central line), upper and lower quartiles (box limits), and min-to-max range. (*, *p* < 0.05).

The association between TMED1 expression and clinical characteristics and survival outcomes in CRC patients was explored. As Figure 2A shows, TMED1 expression was significantly higher in COAD than in READ (*p* < 0.05). The increased expression of TMED1 was significantly correlated with the advanced T stage (*p* < 0.05, Figure 2B) (Table S3). A Sankey diagram was used to visualize the primary tumor sites, T histopathologic stage, TMED1 expression, and survival status in CRC patients (Figure 2C). It was indicated that T4 stage patients mainly belonged to the high expression group, which was finally correlated with poor prognosis in CRC patients. The Kaplan–Meier analysis results showed a negative correlation between different TMED1 expression groups and OS based on TCGA data (*p* < 0.01, Figure 2D). Similar results were acquired for disease-free survival, and increased TMED1 was correlated with a poor prognosis (Figure 2E). The validation of prognostic on the Kaplan–Meier Plotter platform was carried out using an independent cohort (GSE12945, GSE13294, GSE14333, GSE143985, GSE17538, GSE18088, GSE26682, GSE26906, GSE30540, GSE31595, GSE33114, GSE34489, GSE37892, GSE38832, GSE39582, GSE41258) obtained from the GEO database (Figure 2F,G). For COAD and READ patients, we performed subgroup analyses on prognosis. We found that the group with high expression of TMED1 showed a poorer prognosis than the group with low expression (Figure 2H). In addition, T stage and TMED1 expression were combined to explore the prognosis. The result indicated that patients with both high TMED1 expression and T4 stage had the worst prognosis (Figure 2I). These results suggested that increased expression of TMED1 was linked to an unfavorable prognosis in CRC patients.

### 3.3. Knockdown of TMED1 Decreased Proliferation and Induced Apoptosis in CRC Cells

**Figure 3:**
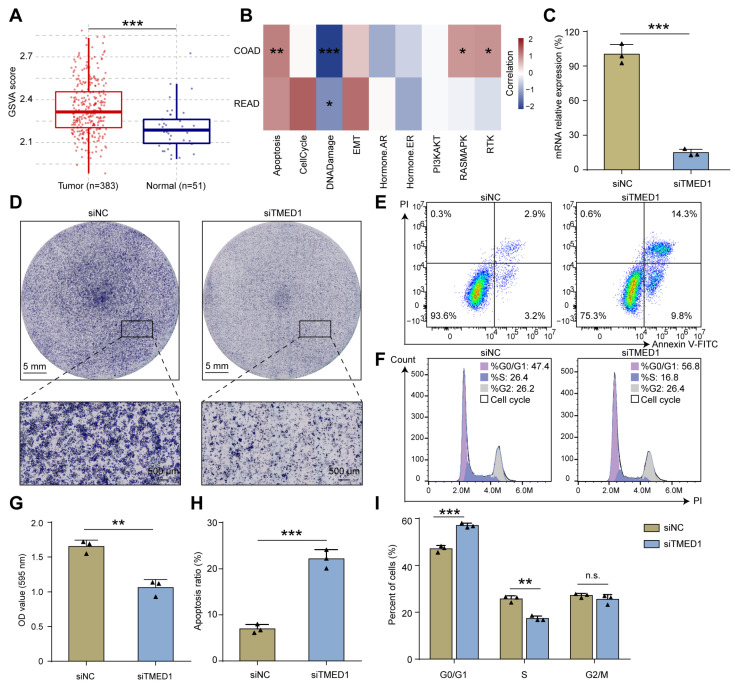
Knockdown of TMED1 decreased proliferation and induced apoptosis in CRC cells. (**A**) GSVA analysis of tumor and normal samples in CRC patients. Boxplots show the median (central line), upper and lower quartiles (box limits), and min-to-max range. (**B**) The association between the GSVA score and the activity of cancer-related pathways in CRC. (**C**) The mRNA expression of TMED1 after transfection (mean ± SD). (**D**) Crystal violet staining of HCT 116 cells after TMED1 knockdown. Flow cytometry detected apoptosis (**E**) and cell cycle (**F**) in HCT 116 cells after TMED1 knockdown. Statistical analysis of crystal violet staining (**G**), apoptosis ratio (**H**), and percentage of cells in the cell cycle (**I**) from at least three independent experiments (mean ± SD). (*, *p* < 0.05; **, *p* < 0.01; ***, *p* < 0.001; n.s., no significance).

Gene set variation analysis (GSVA) demonstrated that TMED1 was associated with apoptosis, cell cycle, epithelial–mesenchymal transition (EMT), and the RAS signal pathway in CRC (Figure 3A,B). Therefore, we further investigated whether TMED1 impacted the aggressive phenotype of CRC. Firstly, TMED1 was effectively silenced by siRNA transfection in the HCT 116 cells, and the knockdown efficiency exceeded 70% (*p* < 0.001, Figure 3C). The results of CCK-8 assay showed that the HCT 116 and RKO cell proliferation were inhibited when TMED1 was knockdown (Figure S2). Then, the crystal violet staining indicated that the knockdown of TMED1 could significantly inhibit CRC cell proliferation (*p* < 0.01, Figure 3D,G). In addition, when TMED1 was knocked down, the apoptosis rate was significantly increased by 15.19% (*p* < 0.001; Figure 3E,H), and the proportion of cells at the G0/G1 stage was significantly (*p* < 0.001) increased while that at the S stage was significantly (*p* < 0.01) decreased (Figure 3F,I). These results indicated that TMED1 might promote the progression of CRC by influencing cell apoptosis and cell cycle regulation.

### 3.4. The Correlation between TMED1 and Immune Infiltration in CRC

**Figure 4:**
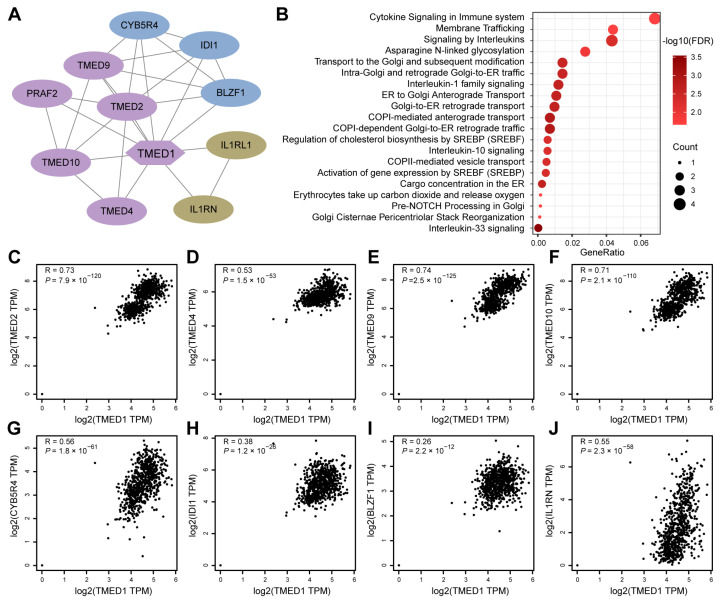
PPI network and functional enrichment analyses. (**A**) The network is established by TMED1 and its co-expression genes. (**B**) Reactome pathway enrichment analyses for the network. (**C**–**J**) The correlation analyses between TMED1 expression and its co-expressed genes in CRC from TCGA and GTEx data on the GEPIA platform.

To gain further insights into the role of TMED1 in CRC, a PPI network was constructed using the STRING database (Figure 4A). A significant correlation of the expression levels between TMED1 and various co-expressed genes was observed, especially among TMED family members such as TMED2, TMED4, TMED9, and TMED10 (Figure 4C–F). Moreover, there was a significant positive correlation between TMED1 and the genes CYB5R4, IDI1, BLZF1, and IL1RN (Figure 4G–J). The 11 genes that co-expressed with TMED1 were analyzed by the Reactome pathway database, which revealed potential functions in “cytokine signaling in the immune system”, “signaling by interleukins”, “interleukin-1 family signaling”, “interleukin-10 signaling”, and “interleukin-33 signaling” (Figure 4B). These results indicated that TMED1 might participate in immune regulation.

**Figure 5:**
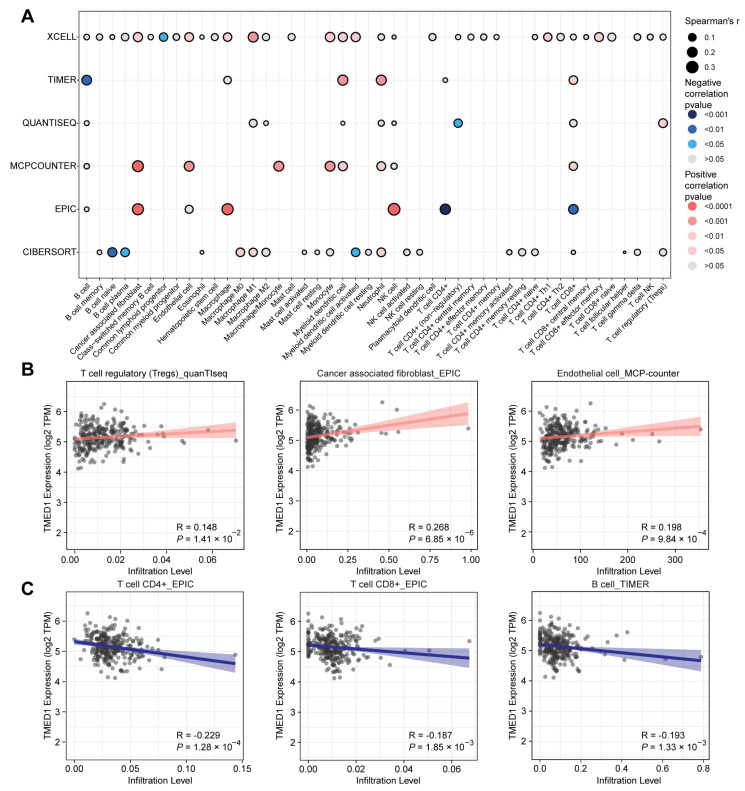
Relationship between TMED1 and immune cell infiltration in CRC. (**A**) Correlation analysis between TMED1 and immune cell infiltration using xCell, TIMER, quanTIseq, MCP-counter, EPIC, and CIBERSORT algorithms, respectively. (**B**) Correlation scatter plot between the infiltration level of immunosuppressive cells and the expression of TMED1 from the TCGA database. (**C**) Correlation scatter plot between the infiltration level of anti-tumor immune cells and the expression of TMED1 from the TCGA database.

Then, Spearman’s correlation analysis was conducted to explore the association between TMED1 expression and the infiltration of immune cells in CRC. These results indicated that TMED1 expression was positively correlated with the infiltrate estimation value of immunosuppressive cells, such as regulatory T cells (Tregs, *p* = 1.41 × 10^−2^), cancer-associated fibroblasts (CAFs, *p* = 6.85 × 10^−6^), and endothelial cells (*p* = 9.84 × 10^−4^) (Figure 5A,B). In contrast, the infiltrate estimation value was negatively correlated with the anti-tumor immune cells, including CD4+ T cells (*p* = 1.28 × 10^−4^), CD8+ T cells (*p* = 1.85 × 10^−3^), and B cells (*p* = 1.33 × 10^−3^) (Figure 5A,C). Subsequently, we investigated the relationship between the expression of TMED1 and immune checkpoint inhibitors and immunostimulators in CRC patients. According to Spearman’s correlation analysis, there were positive correlations observed between the expression of TMED1 and immune checkpoint inhibitors VTCN1 (*p* = 5.90 × 10^−4^) and LGALS9 (*p* = 0.04) in CRC (Figure S1A). In addition, the expression of TMED1 had negative correlations with the immunostimulators, including CD28, CD40LG, HHLA2, and ICOS (all *p* < 0.01, Figure S1B).

### 3.5. TMED1 Increased the Drug Resistance of Cancer Cells

**Figure 6:**
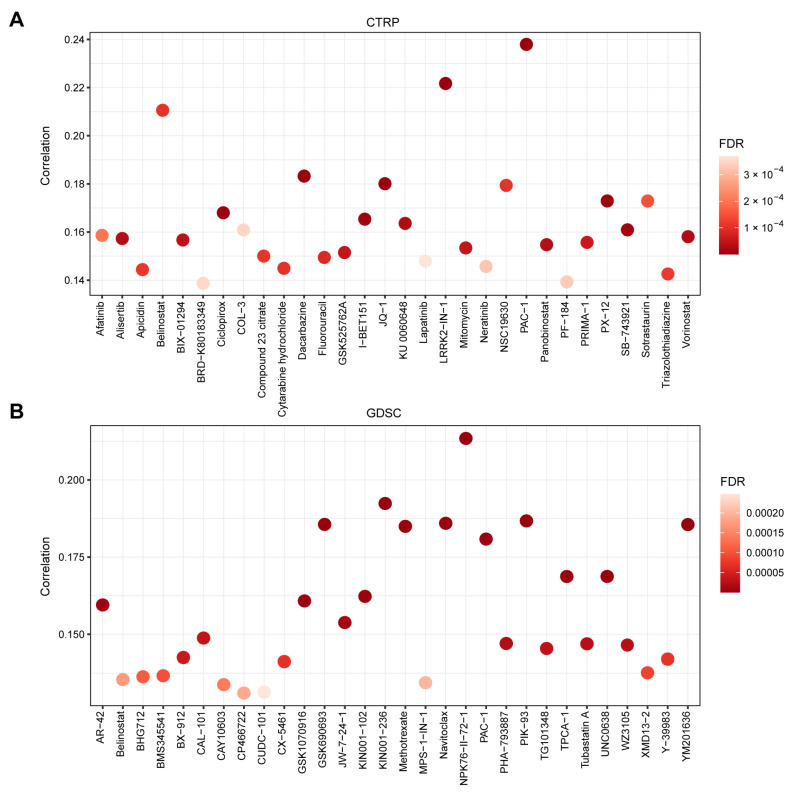
TMED1 increased the drug resistance of cancer cells. (**A**) The correlation between TMED1 expression and the sensitivity (IC_50_) of CTRP drugs (top 30) and (**B**) GDSC drugs (top 30).

At last, the GSCA platform was employed to investigate the correlation between TMED1 expression and drug sensitivity (IC_50_) to characterize its clinical significance. The CTRP and GDSC datasets contain gene expression profiling and drug sensitivity data from numerous cancer cell lines. On the one hand, a dataset consisting of 1001 cancer cell lines and 481 small molecules from the CTRP database was utilized to explore the association between the expression of TMED1 and the IC_50_ values of diverse chemical compounds. Pearson’s correlation analysis revealed a positive correlation between the upregulation of TMED1 and the IC_50_ values of various compounds, including PAC-1, LRRK2-IN-1, belinostat, dacarbazine, JQ-1, and others. The results demonstrated that the elevation of TMED1 could decrease drug sensitivity for the majority of small molecules (Figure 6A). On the other hand, the data from the GDSC database involved 860 cancer cell lines with 265 compounds that were used to investigate the relationship between TMED1 and the IC_50_ of these chemical compounds. Consistent with the above results, TMED1 was correlated with decreased sensitivity to the majority of small molecules, such as NPK76-II-72-1, KIN001-236, PIK-93, navitoclax, GSK690693, and so on (Figure 6B). These results indicated that TMED1 might be a therapeutic target for cancer resistance.

## 4. Discussion

Colorectal cancer is a common type of gastrointestinal tumor, and primary treatments for malignant CRC may not be universally effective for all patients [52]. In light of this concern, there is an urgent need for the identification of a potential biomarker and therapeutic target that can effectively serve as a prognostic indicator. As the critical regulators of protein trafficking in the secretory pathway, TMED family proteins are involved in developing multiple cancers [53]. TMED family proteins are involved in protein transport and secretion, which are critical processes for the growth and survival of cancer cells [15]. Additionally, some TMED proteins have been shown to regulate signaling pathways that promote tumor growth and survival, such as the Wnt signaling pathway and the PI3K/AKT signaling pathway [21,54]. TMED1 belongs to the TMED protein family and is overexpressed in various cancers [11,18]. However, the biological behavior of TMED1 in malignant tumors has yet to be fully elucidated, especially in CRC. In this study, the expression of TMED1 and the association with the tumor’s primary location, as well as the T stage, were analyzed. Additionally, this is the first study to explore the prognostic significance and potential biological function of TMED1 in the progression of CRC.

In this study, it was found that TMED1 expression substantially increased in CRC tumor tissues compared to normal tissues in the TCGA, GEO, and CPTAC datasets. Consistently, HPA immunohistochemistry staining data indicated a lower expression of TMED1 in the normal colon than in the CRC. Moreover, TMED1 was differentially expressed in different T stages and was higher in COAD than READ. In addition, the analysis of Kaplan–Meier plots found that elevated TMED1 expression was notably correlated with an unfavorable prognosis in CRC patients. The role of TMED1 in tumors, especially in CRC, has been explored rarely. To further explore the functions of TMED1, a series of cell experiments were performed. In HCT 116 cells, cell apoptosis was induced, and the cell transformation from the G0/G1 phase to the S phase was also inhibited after knocking down TMED1. Based on these findings, TMED1 may be a novel tumor oncogene and prognostic biomarker for CRC.

The tumor immune microenvironment is very important for the occurrence and development of malignant tumors [55], and the alterations in immune infiltrate have an important influence on overall survival outcomes [56]. Few studies have reported the immune regulation function of TMED1, especially in malignant tumors. It was found that TMED1 was required for IL-33-induced IL-8 and IL-6 production through interaction with IL-1R family member ST2L [57]. In addition, TMED1 formed a complex with the ubiquitin–protein ligases RNF26, TMEM43, ENDOD1, and TMEM33 to modulate innate immune signaling through the cGAS-STING pathway [58]. These results indicated that TMED1 may play an essential role in immune signaling. In our study, Reactome pathway analysis showed that immune system-related signaling pathways were significantly enriched, such as “cytokine signaling in the immune system,” “signaling by interleukins,” “interleukin-1 family signaling,” “interleukin-10 signaling,” and “interleukin-33 signaling.” Therefore, it is essential to analyze the association between TMED1 expression and immune infiltration. Tumor immune cells play a crucial role in the regulation of immune responses within tumors [59]. Emerging evidence indicates that various components of the immune system, including regulatory T cells [60], cancer-associated fibroblasts [61], and endothelial cells [62], play an important role in promoting tumor progression within the tumor microenvironment. Our findings showed that TMED1 expression was positively correlated with Tregs, cancer-associated fibroblasts, and endothelial cells. Recent research highlights the significance of both CD4+ T cells, CD8+ T cells, and B cells in the field of cancer immunotherapy [63,64]. According to the bioinformatics analysis, we found a negative correlation between TMED1 expression and CD4+ T cells, CD8+ T cells, and B cells. Immune checkpoint inhibitors have gained much attention in the landscape of cancer immunotherapy [65]. In this study, TMED1 was positively correlated with immune checkpoint inhibitors (VTCN1 and LGALS9) and had negative correlations with immunostimulators (CD28, CD40LG, HHLA2, and ICOS). Taken together, TMED1 exhibited a close association with immune infiltration, emphasizing the need for further investigation in this area.

It is well-known that drug resistance is a crucial influence factor for cancer therapy [66]. To explore the impact of TMED1 expression on drug sensitivity, Pearson’s correlation analysis was performed. In our study, the drug sensitivity analysis revealed a positive correlation between the IC_50_ values of various chemical compounds and TMED1 expression. Among these compounds, PAC-1 was found in both the CTRP and GDSC datasets. It was reported that PAC-1 acts as a procaspase-3 activator that induces apoptosis in cancer cells [67]. Moreover, phase I clinical studies indicated that PAC-1 could treat advanced malignant tumors [68]. The above results suggested that TMED1 might be a potential cancer-resistant therapeutic target; however, further experiments need to be performed to verify these findings.

Nevertheless, the current study has several limitations. Firstly, the high expression of TMED1 and poor prognosis analysis were explored based on public databases; it would be more meaningful to be validated by a Chinese cohort. Secondly, the signaling pathways that TMED1 influenced needed to be clarified, which might be explored by transcriptomics or proteomics. Thirdly, further experiments in vitro and in vivo should be performed to prove the impact of TMED1 on the growth of colorectal cancer and clarify the specific mechanism.

## 5. Conclusions

In this study, multi-omics analyses reveal TMED1 is highly expressed in CRC and associated with poor prognosis. TMED1 may promote the progression of CRC by regulating cell apoptosis and the cell cycle, influencing immune regulation, and increasing drug resistance. In brief, TMED1 can serve as a promising therapeutic target and an unfavorable prognostic biomarker for colorectal cancer.

## Data Availability

Data are contained within the article and Supplementary Materials.

## Supplementary Materials

The following supporting information can be downloaded at: https://www.mdpi.com/article/10.3390/biology13020083/s1, Figure S1: Association between TMED1 expression and immunomodulators. (A) The correlation analysis between the expression of TMED1 and immune checkpoint inhibitors (VTCN1 and LGALS9). (B) Correlation scatter plot between the immunostimulator (CD28, CD40LG, HHLA2, and ICOS) and the expression of TMED1. Figure S2: Knockdown of TMED1 decreases the cell proliferation of HCT 116 (A) and RKO cells (B). The mRNA expression of TMED1 after transfection with siRNA in HCT 116 (C) and RKO cells (D). Table S1: The sequence of oligonucleotides for cell transfection. Table S2: The sequence of primers for qPCR. Table S3: TMED1 expression in different tumor sites and T stages.
