# Supplementary figures and images for: Integrative Multi-Omics Analysis Identifies Transmembrane p24 Trafficking Protein 1 (TMED1) as a Potential Prognostic Marker in Colorectal Cancer
Source: Biology (Basel). 2024 Jan 29;13(2):83. doi: 10.3390/biology13020083 (PMC10886729; doi:10.3390/biology13020083)

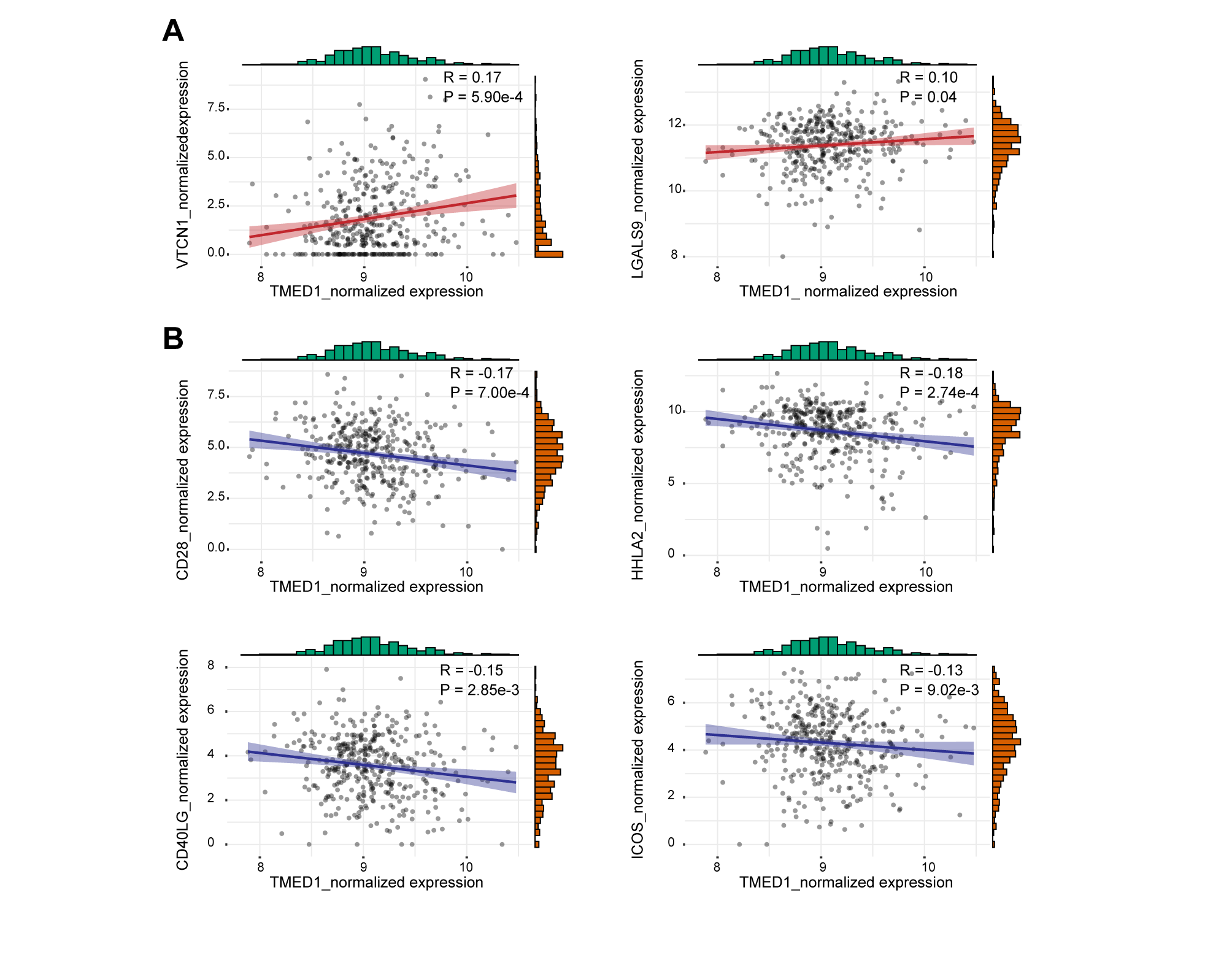

Supplement: Supplementary file 1 [file biology-13-00083-s001.zip › Figure S1.tif]

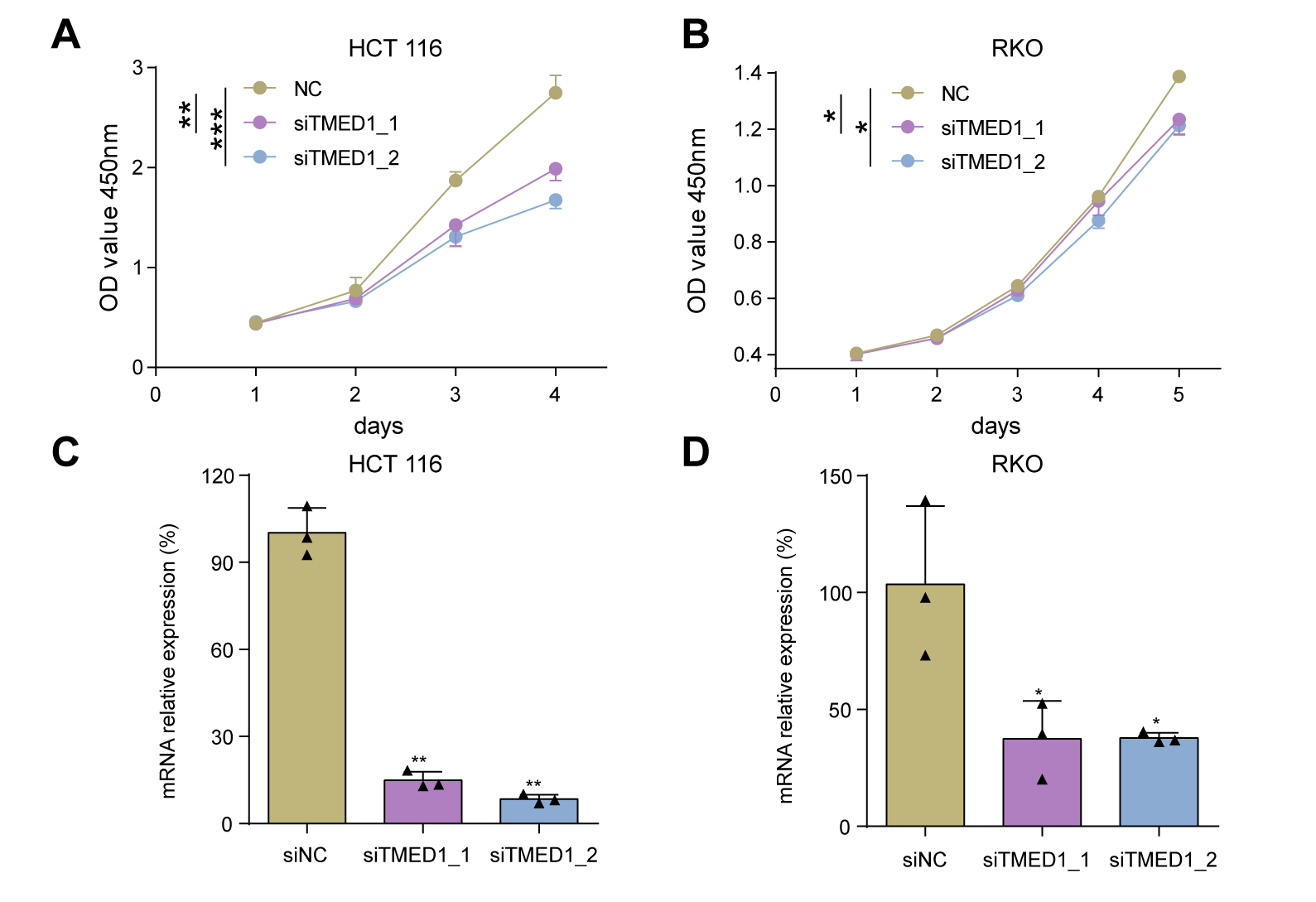

Supplement: Supplementary file 1 [file biology-13-00083-s001.zip › Figure S2.tif]
